# Tumor-targeting novel manganese complex induces ROS-mediated apoptotic and autophagic cancer cell death

**DOI:** 10.3892/ijmm.2015.2073

**Published:** 2015-01-20

**Authors:** JIA LIU, WENJIE GUO, JING LI, XIANG LI, JI GENG, QIUYUN CHEN, JING GAO

**Affiliations:** 1 School of Pharmacy and Chemical Engineering, Jiangsu University, Zhenjiang, Jiangsu 212013, P.R. China; 2 School of Chemistry and Chemical Engineering, Jiangsu University, Zhenjiang, Jiangsu 212013, P.R. China

**Keywords:** manganese complex, anticancer, autophagy, apoptosis, reactive oxygen species

## Abstract

In this study, the antitumor activity of the novel manganese (II) compound, Adpa-Mn {[(Adpa)Mn(Cl)(H_2_O)] (Adpa=bis(2-pyridylmethyl)amino-2-propionic acid)}, and its possible mechanisms of action were investigated. *In vitro*, the growth inhibitory effects of Adpa-Mn (with IC_50_ values lower than 15 *μ*M) on tumor cell lines were examined by MTT assay. We found that this compound was more selective against cancer cells than the popular chemotherapeutic reagent, cisplatin. We then found that Adpa-Mn achieved its selectivity against cancer cells through the transferrin (Tf)-transferrin receptor (TfR) system, which is highly expressed in tumor cells. Furthermore, Adpa-Mn induced both apoptosis and autophagy, as indicated by chromatin condensation, the activation of poly(ADP-ribose) polymerase (PARP), Annexin V/prop-idium iodide staining, an enhanced fluorescence intensity of monodansylcadaverine (MDC), as well as the elevated expression of the autophagy-related protein, microtubule-associated protein 1 light chain 3 (LC3). In addition, Adpa-Mn induced the generation of intracellular reactive oxygen species (ROS) and its anticancer effects were significantly reduced following pre-treatment with the antioxidant, N-acetyl cysteine, indicating that ROS triggered cell death. *In vivo*, the induction of apoptosis and autophagy in tumor tissue was confirmed following treatment with Adpa-Mn, which contributed to its significant antitumor activity against hepatocellular carcinoma (Hep-A cell) xenografts at 10 mg/kg. Taken together, these data suggest the possible use of Adpa-Mn as a novel anticancer drug.

## Introduction

Metal ions are required for a number of critical functions in living organisms and are becoming increasingly important as diagnostic and therapeutic tools in the study and and treatment of a variety of human diseases (1,2). Since the success of cisplatin, increasing attention has been paid to metal complexes, which has been one of the most rapidly developing areas of anticancer drugs (3-9). It has been demonstrated that a number of bioinorganic complexes contain metals, such as iron (Fe), ruthenium (Ru) and can trigger reactive oxygen species (ROS)-mediated cell death (10).

Manganese (Mn) is a widely distributed metal which is a required co-factor for many ubiquitous enzymes (11). It has been proven that Mn(II) ions are mainly absorbed and transported by the transferrin (Tf)-transferrin receptor (TfR) system (12,13). Studies have also demonstrated that TfR is highly expressed in some types of tumor tissue (14–16). To date, simple Mn(II) salts have been reported to exert anti-proliferate effects on several cancer cell lines (17,18) and Mn(II) complexes containing thiosemicarbazone or hydrazone groups have been reported as antitumor agents (19), generally by the induction of apoptotic cell death at rather high concentrations (in the mM range). Based on these data, we designed and synthesized Mn(II)-containing compounds, in an aim to develop novel tumor-targeting lead chemotherapeutic agents. During our research, a novel Mn(II) complex was synthesized and characterized, and its anticancer activity was previously investigated in a previous study of ours (20); however, its mechanisms of action remain unclear.

Of note, the majority antitumor therapies, including chemotherapy primarily act by inducing apoptosis (type I programmed cell death) in cancer cells; thus, defects in the apoptotic programs may cause resistance to the therapeutic agents (21,22). Thus, an alternative cell death pathway termed autophagic cell death (type II programmed cell death) has emerged as an important mechanism of cancer cell death induced by chemotherapeutic agents (type II programmed cell death) (10,23,24). Autophagy can act as a pro-death mechanism which leads to the destruction of cancer cells, since autophagy is a ‘self-cannibalistic’ process. There is evidence that autophagy is required for the death of cancer cells with defects in apoptosis (25,26). Moreover, new insights into the molecular mechanisms of autophagy are now leading to the discovery of exciting new potential drug targets (27) and researchers have pointed out that apoptosis and autophagy are tightly connected and may be regulated by the same trigger, such as ROS (28,29).

In the present study, we demonstrate that the novel manganese (II) compound, Adpa-Mn {[(Adpa)Mn(Cl)(H_2_O)] (Adpa=bis(2-pyridylmethyl)amino-2-propionic acid)}, is a promising new anticancer agent which exerts potent selective activity against a wide range of tumor cell lines *in vitro* and a carcinoma xenograft model *in vivo*. Moreover, our results verify that Adpa-Mn causes both apoptotic and autophagic cancer cell death through the induction of ROS generation.

## Materials and methods

### Materials

The compound, Adpa-Mn, was synthesized by Professor Chen Qiuyun. Cyclophosphamide (CTX) was produced by Jiangsu Hengrui Medicine Co., Ltd. (Jiangsu, China). 3-(4,5-Dimethyl-2-thiazolyl)-2,5-diphenyl-2H-tetrazolium bromide (MTT) was purchased from Amresco LLC (Solon, OH, USA) and Annexin V/PI kits for the detection of apoptosis were from Life Technologies (Carlsbad, CA, USA). Culture medium (DMEM/1640), trypsin and EDTA-2·Na were purchased from Thermo Fisher Scientific (Waltham, MA, USA). Fetal bovine serum was obtained from Sijiqing Biological Engineering Materials (Hangzhou, China). The 2',7' dichlorofluorescin diacetate (DCFH-DA) kit was purchased from the Beyotime Institute of Biotechnology (Nantong, China). Antibodies against microtubule-associated protein 1 light chain 3 (LC3; 2775), poly(ADP-ribose) polymerase (PARP; 9542), autophagy-related protein (ATG)7 (2631) and ATG siRNA (6604) were obtained from Cell Signaling Technology (Boston, MA, USA); β-actin (sc-8423), GAPDH (sc-25778), cytochrome *c* (sc-13561), COX IV (sc-376731), tubulin (sc-5546), TfR1 (sc-9099) and caspase-3 (sc-98785) were from Santa Cruz Biotechnology, Inc. (Santa Cruz, CA, USA). 3-Methyladenine (3-MA), N-acetyl cysteine (NAC), chloroquine (CQ), cisplatin, ferric citrate and deferoxamine (DFO) were purchased from Sigma-Aldrich. Wortmanin was purchased from Beyotime. All other chemicals were of high purity and were from commercial sources.

### Cell culture

The human cancer cell lines, including HeLa (cervical adenocarcinoma), HepG2 (hepatocellular carcinoma), A549 (lung adenocarcinoma), MCF-7 (breast cancer), U251 (glioblastoma), LoVo (colon cancer), A875 (melanoma) and ECA-109 (human esophageal squamous carcinoma) cells, as well as the human normal liver cell line, WRL-68 (immortalized), were obtained from the Cancer Cell Repository (Shanghai Cell Bank, Shanghai, China). The cells were maintained in DMEM medium supplemented with 10% (v/v) heat-inactivated fetal bovine serum, antibiotics (100 U/ml penicillin and 100 U/ml streptomycin), at 37°C in a humidified atmosphere of 5% CO_2_ (Thermo Fisher Scientific).

### Animals

Female imprinting control region (ICR) mice (6–8 weeks old) were purchased from the Comparative Medicine Research Center of Yangzhou University [Yangzhou, China, register no: SCXK (JIANGSU) 2007-0001]. The mice were maintained on a standard diet and water was made freely available.

### Ethics statement

Animal welfare and experimental procedures were carried out strictly in accordance with the Guide for the Care and Use of Laboratory Animals (The Ministry of Science and Technology of China, 2006) and the related ethical regulations of our university. All efforts were made to minimize the suffering of the animals and to reduce the number of animals used.

### Histological analysis

For histological morphometry, tumor tissues were fixed with 10% formalin and embedded in paraffin, and cut into 5-*μ*m-thick sections and stained with hematoxylin and eosin (H&E; Nanjing Jiancheng Technology Co., Nanjing, China).

### Cell viability assay

The cells were plated at a density of approximately 4 × 10^3^ viable cells per well in 96-well plates. Various concentrations of the compound were used to treat the cells in triplicate. Following incubation for the indicated periods of time, MTT assay was performed to measure cell viability using a 96-well plate reader (Spectra Max 190; Molecular Devices Corp., Sunnyvale, CA, USA).

### Cell morphological changes observed under a Nikon TE2000 microscope

The morphological changes of the H_2_B-GFP-labeled HeLa cells (stable cell line) were observed under a Nikon TE2000 microscope (Nikon, Tokyo, Japan) with a live cell system (LCS) which can provide CO_2_, temperature control and position fixing. The H_2_B-GFP-labeled HepG2 cells, which were incubated with 20 *μ*M Adpa-Mn, were observed for 24 h. The bright and fluorescence imaginations of the cells were recorded and analyzed.

### Cell apoptosis assay

The cells were stained with Annexin V/PI at room temperature for 15 min in the dark and then analyzed using a FACSCalibur flow cytometer (Becton-Dickinson, Franklin Lakes, NJ, USA). Annexin V^+^/PI^−^ and Annexin V^+^/PI^+^ cells were considered as apoptotic cells in the early and late phase.

### Visualization of monodansylcadaverine (MDC)-labeled vacuoles

Autophagic vacuoles were labeled with MDC by incubating the HepG2 cells which were grown on coverslips with 0.05 mM MDC in PBS at 37°C for 10 min. The cellular fluorescent changes were observed under a fluorescence microscope (Nikon; excitation, 380 to 420 nm; emission, 450 nm; Nikon).

### GFP-LC3 plasmid transfection

The HepG2 cells transfected with green fluorescent protein (GFP)-LC3-expressing plasmid were treated with Adpa-Mn, and the fluorescence of GFP-LC3 was viewed under a fluorescence microscope (Nikon).

### Western blot analysis

Proteins were extracted in lysis buffer (30 mM Tris, pH 7.5, 150 mM sodium chloride, 1 mM phenylmethanesulfonyl fluoride, 1 mM sodium orthovanadate, 1% Nonidet P-40, 10% glycerol, and phosphatase and protease inhibitors), separated by SDS-PAGE and electrophcoretically transferred onto polyvinylidene fluoride membranes. The membranes were probed with antibodies (LC3, PARP, ATG7 and β-actin) overnight at 4°C, and then incubated with a horse radish peroxidase-coupled secondary antibody. Detection was performed using a LumiGLO chemiluminescent substrate system [Kirkegaard & Perry Laboratories, Inc. (KPL), Gaithersburg, MD, USA].

### Mitochondrial membrane potential assay

Changes in mitochondrial membrane potential were measured using JC-1 staining. Briefly, following treatment, the HepG2 cells were washed with PBS and incubated with 5 *μ*g/ml JC-1 at 37°C for 30 min. The cells were then washed twice with PBS and immediately assessed by fluorescence spectrometry (Spectra MaxGemini; Molecular Devices Corp.). A 488 nm filter was used for the excitation of JC-1. Emission filters of 535 and 595 nm were used to quantify the population of mitochondria with green (JC-1 monomers) and red (JC-1 aggregates) fluorescence. The ratio of red/green was used to reflect the mitochondrial membrane potential.

### Measurement of intracellular ROS production

The intracellular generation of ROS was analyzed using the probe, DCFH-DA. Cells were incubated with 10 *μ*M DCFH-DA at 37°C for 15 min. The DCF fluorescence distribution of 1×10^4^ cells was tehn measured by fluorescence spectrometry (Spectra MaxGemini; Molecular Devices Corp.) at an excitation wavelength of 488 nm and at an emission wavelength of 535 nm.

### Evaluation of the antitumor effects of Adpa-Mn in vivo

Mouse hepatocellular carcinoma (Hep-A; 1×10^7^) cells (grown in donor mice) were transplanted subcutaneously into the armpits of the ICR mice. One day following transplantion, the mice were randomly allocated to either the control (vehicle control, received PBS) or treatment groups, with 10 mice in each group. The drugs (Adpa-Mn and CTX) were administered intraperitoneally on days 0–9. All efforts were made to minimize the suffering of the animals and to reduce the number of animals used and the mice were sacrificed by cervical dislocation. After the mice were sacrificed, the solid tumors were separated. Tumor weights were measured and the tumor growth inhibition ratio was calculated. The toxic effects of Adpa-Mn on the spleen were also observed.

### Statistical analysis

Comparisons were made by one-way analysis of variance (ANOVA). Differences were considered statistically significant when p<0.05. All experiments were repeated at least 3 times. All graphs were created using GraphPad Prism 5 software.

## Results

### Adpa-Mn exerts inhibitory effects on various types of cancer cells

**Figure 1:**
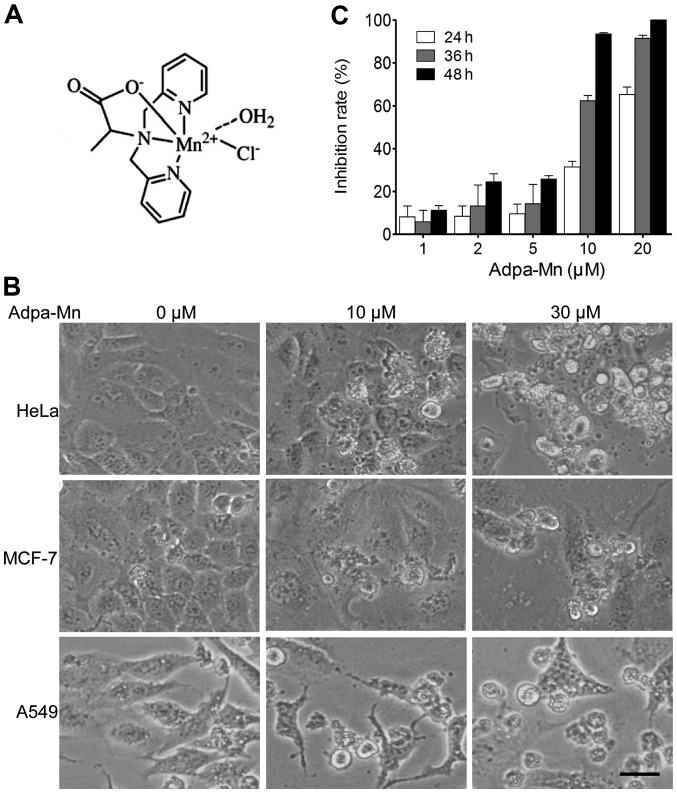
Adpa-Mn significantly inhibits the proliferation of various types of human cancer cells. (A) Structure of Adpa-Man. HepG2 cells were exposed to various concentrations of Adpa-Mn. (B) The morphological changes (cell body shrinkage and cell number reduction) of the cells were photographed. (C) Cell proliferation was determined by MTT assay every 12 h. Data represent the means ± SD of 3 different experiments.

First we examined the cytotoxic effects of Adpa-Mn (chemical structure shown in Fig. 1A) on various types of human cancer cells, including human cervical cancer cells (HeLa), human hepatocellular carcinoma cells (HepG2), human lung cancer cells (A549), human breast cancer cells (MCF-7), human glioblastoma cells (U251), human colon cancer cells (LoVo), human melanoma cells (A875) and human esophageal squamous carcinoma cells (ECA-109). The morphological changes (cell body shrinkage and cell number reduction) of the cells were photographed (Fig. 1B) and the 50% inhibitory concentration (IC_50_) was calculated (Table I). Our results revealed that Adpa-Mn exerted a significant cytotoxic effect with an IC_50_ between 5 and 20 *μ*M. Furthermore, we demonstrated that Adpa-Mn inhibited HepG2 cell proliferation not only in a dose-dependent manner, but also in a time-dependent manner (Fig. 1C).

### Adpa-Mn selectively kills cancer cells through the Tf-TfR system

**Figure 2:**
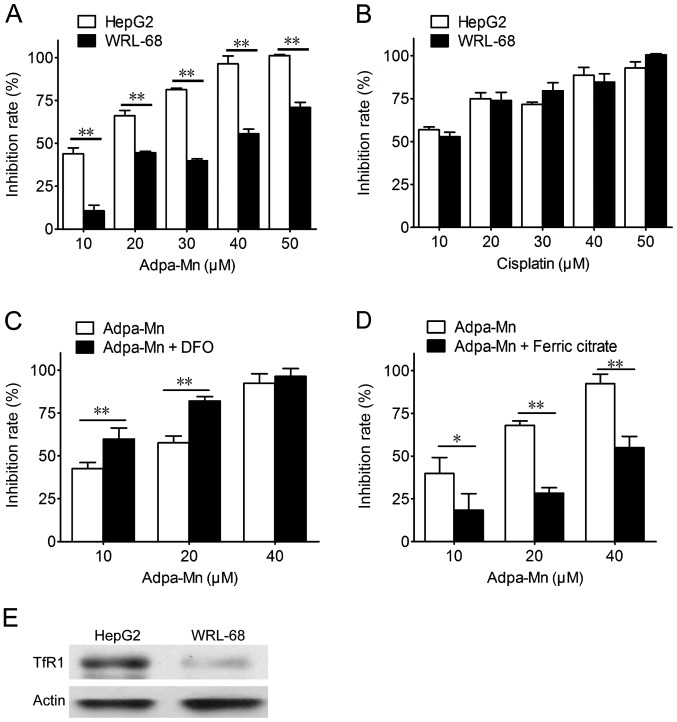
Adpa-Mn exhibits selective inhibition on cancer cell proliferation through the transferrin/transferrin receptor (Tf-1/TfR-1) system. HepG2 cells and WRL-68 cells were treated with various concentrations of (A) Adpa-Mn or (B) cisplatin for 24 h and cell viability was detected by MTT assay. HepG2 cells were pre-treated with deferoxamine (DFO; 10 *μ*M) (C) or ferric citrate (100 *μ*M) (D) for 24 h, then treated with various concentrations of Adpa-Mn for 24 h. (E) The expression of TfR-1 in HepG2 and WRL-68 cells. Data represent the means ± SEM of 3 different experiments. ^*^p<0.05 and ^**^p<0.01 vs. the respective control.

The selectivity of Adpa-Mn on cancer cells was examined. Adpa-Mn demonstrated significant selectivity towards the liver cancer cells (HepG2) compared with the non-malignant liver epithelial cells (WRL-68) (Fig. 2A), and compared to treatment with cisplatin (Fig. 2B). The preferential toxicities toward the cancer cells compared to the non-cancer cells suggest the possibile use of this compound as an antitumor agent. Due to the high expression level of TfR in the tumor cells, the selectivity of Adpa-Mn may be due to its transport mechanisms. As shown in Fig. 2E, the expression of TfR1 in the HepG2 cells was higher than that in the WRL-68 cells. As shown in Fig. 2C and D, pre-treatment with ferric citrate reduced the inhibitory effects of Adpa-Mn on the growth of HepG2 cells, while pre-treatment with deferoxamine (DFO) promoted them.

### Adpa-Mn induces apoptotic cell death through the mitochondrial pathway

**Figure 3:**
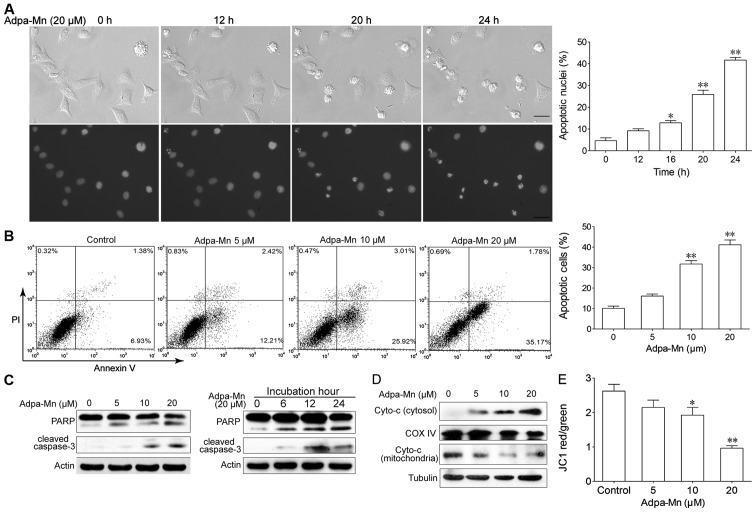
(Adpa-Mn induces apoptotic cell death. (A) H_2_B-GFP-labeled HeLa cells (stable cell line) were treated with Adpa-Man (20 *μ*M) for 24 h, morphology and nuclei were photographed using a Nikon TE2000 microscope with a live cell system. Apoptotic nuclei were counted in 10 fields of vision at each time point and the apoptotic percentage was calculated. Scale bar, 5 *μ*m. (B) HepG2 cells treated with Adpa-Mn were collected and subjected to Annexin V/propidium iodide (PI) staining. (C) PARP and caspase-3 activation were assessed by western blot analysis. (D) The release level of cytochrome *c* from the mitochondria was examined by western blot analyiss. (E) HepG2 cells treated with Adpa-Mn were collected and subjected to mitochondrial membrane potential analysis. Data represent the means ± SD of 3 different experiments. ^*^p<0.05 and ^**^p<0.01, as compared with the untreated (control) group.

We then wished to determine which cell death pathway was employed when the cells were treated with Adpa-Mn. The possibility of apoptosis was investigated. As shown in Fig. 3A, following incubation with Adpa-Mn for 12 h, cell shrinkage and chromatin condensation were observed. As the inubation time increased, the nuclei became condensed and had divided into several parts; apoptotic bodies had emerged, and an increasing number of cells began to exhibit these characteristics. We calculated the percentage of cells which showed these characteristics and found that this percentage increased in a time-dependent manner (Fig. 3A). FACS analyses revealed that the number of Annexin V^+^/PI^−^ cells had increased from 6.9 to 25.9% (10 *μ*M) and from 35.2% (20 *μ*M) following treatment with Adpa-Mn (Fig. 3B). Western blot analysis revealed that Adpa-Mn triggered the activation of PARP and the cleavage of caspase-3 (Fig. 3C) in a dose- and time-dependent manner.

To further examine the pathway of apoptosis, we monitored the changes in apoptotic molecules related to the mitochondrial pathway in the HepG2 cells. As shown in Fig. 3D and E, treatment with Adpa-Mn disrupted the mitochondrial trans-membrane potential and with the collapse of the mitochondrial transmembrane potential, the release of cytochrome *c* from the mitochondrion to the cytosol was greatly increased in a dose-dependent manner (Fig. 3C). These results indicate that the mitochondrial apoptotic pathway is involved in the Adpa-Mn-induced apoptosis of cancer cells.

### Adpa-Mn induces autophagic cell death

**Figure 4:**
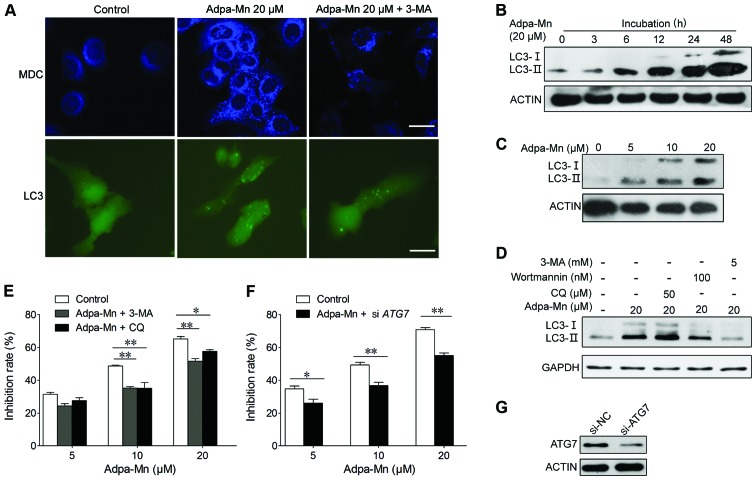
Adpa-Mn induces autophagic cell death. (A) HepG2 cells transfected with GFP-LC3 cDNA were treated with 20 *μ*M Adpa-Mn for 12 h with or without pre-treatment with 5 mM 3-methyladenine (3-MA) for 2 h. The formation of vacuoles containing GFP-LC3 (dots) was examined by fluorescence microscopy. In another set of experiments, HepG2 cells were treated with 20 *μ*M Adpa-Mn for 12 h with or without pre-treatment with 5 mM 3-MA for 2 h and then incubated with 0.05 mM monodansylcadaverine (MDC) for 10 min. The cells were then analyzed by fluorescence microscopy. Scale bar, 5 *μ*m. Protein expression of LC3 in (B-D) was determined by western blot analysis. (B) HepG2 cells were cultured with 20 *μ*M Adpa-Mn for 3, 6, 12, 24 and 48 h. (C) HepG2 cells were cultured with the indicated concentrations of Adpa-Mn for 24 h. (D) HepG2 cells were treated with 20 *μ*M Adpa-Mn for 24 h with or without 3-MA, wartmanin and chloroquine (CQ) pre-treatment for 2 h. (E) HepG2 cells were treated with 20 *μ*M Adpa-Mn for 24 h with or without 3-MA, and CQ pre-treatment for 2 h. MTT assay was used to evaluate the cell death rate. (F) HepG2 cells were transfected with control siRNA or siRNA targeting autophagy-related gene (ATG7). After 48 h, the cells were treated with 0, 5, 10 or 20 *μ*M Adpa-Mn for 24 h, and cell death was measured by MTT assay. (G) The knockdown of ATG7 was confirmed by western blot analysis. Data represent the means ± SEM of 3 different experiments. ^*^p<0.05 and ^**^p<0.01 vs. respective control.

We also wished to determine whether autophagic cell death contributes to the cytotoxic effects of Adpa-Mn. The possibility of the induction of autophagy was analyzed by autophagic vacuole organelle (AVO) formation, the formation of GFP-LC3 vacuoles and LC3 conversion. AVO formation was detected and measured by staining with MDC, as previously described (30). The Adpa-Mn-treated HepG2 cells showed a greater fluorescence intensity and a greater number of MDC-labeled particles compared with the control (untreated) group (Fig. 4A), indicating that Adpa-Mn increased MDC recruitment to autophagosomes in the cytoplasm which was suppressed by the autophagy inhibitor, 3-MA (Fig. 4A).

Conversion of LC3-I (19 kDa) to the pre-autophagosomal and autophagosomal membrane-bound form of LC3-II (17 kDa) is another specific marker of autophagosome formation (31). GFP-fused LC3 was transfected into the cells to detect autophagy. As shown in Fig. 4B, the formation of GFP-LC3-labeled vacuoles in the HepG2 cells was markedly increased 12 h following treatment with 20 *μ*M Adpa-Mn. The formation of these vacuoles was inhibited by treatment with 3-MA, a specific inhibitor of the autophagic process during the early stages (Fig. 4A). Consistent with the above results, the LC3-I to LC3-II conversion markedly increased with the increasing incubation time or the increasing dose of Adpa-Mn in the HepG2 cells, as shown by western blot analysis (Fig. 4B and C); this was also inhibited by treatment with the autophagy inhibitors, wortmanin and 3-MA (Fig. 4D).

To determine whether autophagy is associated with the cell death induced by Adpa-Mn in the HepG2 cells, we examined whether the inhibition of autophagy using autophagy inhibitors or by silencing autophagy-related genes affects cell death in the HepG2 cells. First, using MTT assay, we found that pre-treatment with the autophagy inhibitor, 3-MA, or the autophagolysosome fusion inhibitor, CQ, significantly inhibited Adpa-Mn-induced cell death in a dose-dependent manner (Fig. 4E). Second, we also found that silencing autophagy-related genes (ATG7) using siRNA significantly reduced the cell death induced by Adpa-Mn (Fig. 4F and G). These results suggest that autophagy contributes to the death of HepG2 cells treated with Adpa-Mn.

### ROS generation triggered by Adpa-Mn is indispensable for the induction of apoptosis and autophagy

**Figure 5:**
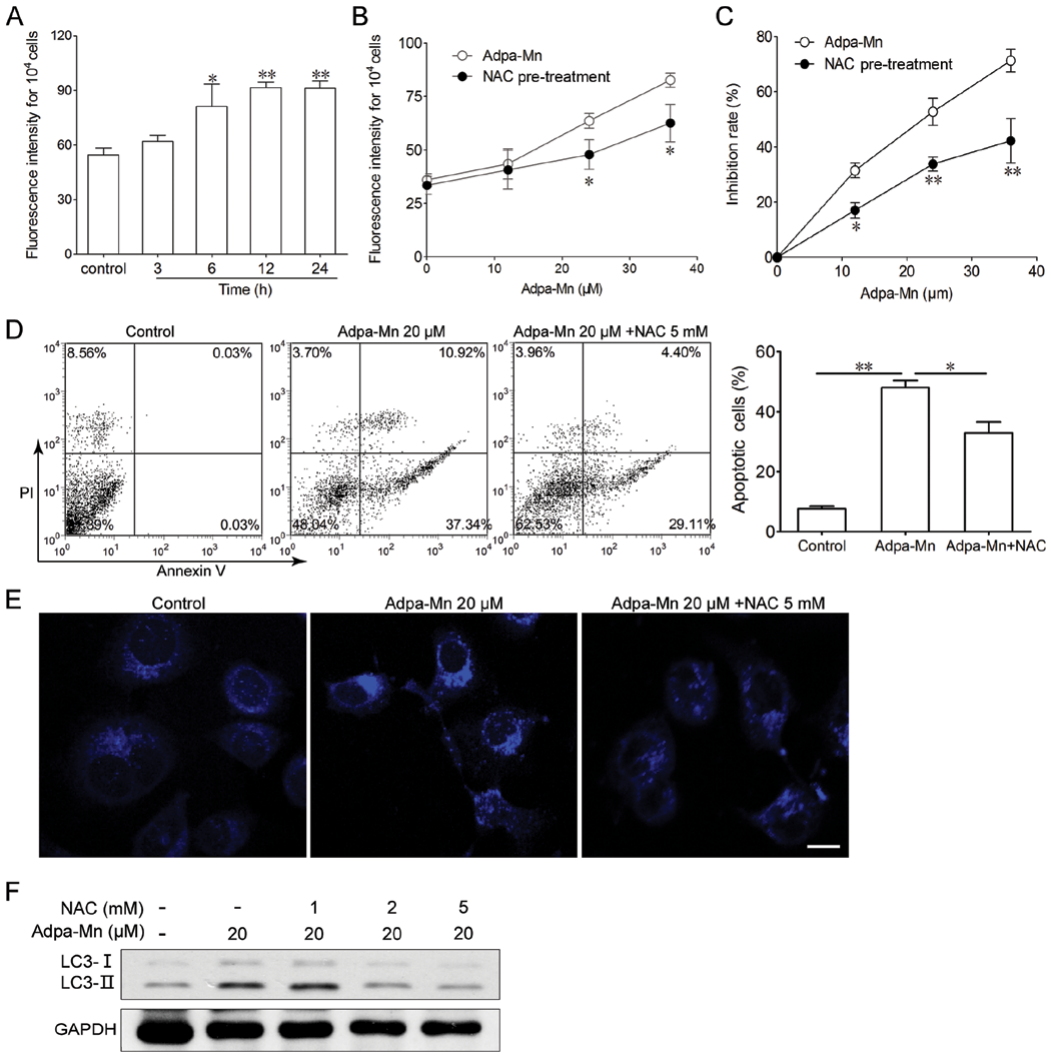
Adpa-Mn induces apoptotic and autophagic cell death dependent on reactive oxygen species (ROS) generation. (A) HepG2 cells were exposed to 20 *μ*M Adpa-Mn for different periods of time and ROS generation was detected by DCFH-DA. (B) HepG2 cells were exposed to 5, 10, 20 *μ*M Adpa-Mn for 12 h with or without NAC pre-treatment for 2 h and ROS generation was detected by DCFH-DA. (C) HepG2 cells were exposed to 5, 10, 20 *μ*M Adpa-Mn for 24 h with or without NAC pre-treatment for 2 h and cell death was examined by MTT assay. (D) HepG2 cells were exposed to 20 *μ*M Adpa-Mn for 24 h with or without NAC pre-treatment for 2 h and apoptosis was evaluated by Annexin V/propidium iodide (PI) staining. (E and F) HepG2 cells were exposed to 20 *μ*M Adpa-Mn for 12 h with or without NAC pre-treatment for 2 h and autophagic vacuole organelle (AVO) formation and LC3 expression were evaluated by western blot analysis. Scale bar, 5 *μ*m.

To determine whether ROS play an important role in the cell death induced by Adpa-Mn, the intracellular ROS levels were measured by fluorescence spectrometry after the cells were labeled with DCFH-DA. As shown in Fig. 5A, treatment with 20 *μ*M of Adpa-Mn for 6 h led to an increase in ROS generation in the HepG2 cells. The generation of ROS significantly increased, as detected by the higher fluorescence intensity compared to the control (untreated) group. The generation of ROS increased in a dose- and time-dependent manner, suggesting that the continuous generation of ROS is involved in the whole process of Adpa-Mn-induced cell death (Fig. 5A). To determine the role of ROS in the Adpa-Mn-induced cell death, we examined whether the inhibition of ROS by NAC affects apoptosis or autophagy in HepG2 cells. As shown in Fig. 5B, pre-treatment with NAC effectively suppressed the generation of ROS. MTT assay revealed that the cell death induced by Adpa-Mn was markedly reduced by NAC (Fig. 5C). Annexin V/PI staining also revealed that pre-treatment with NAC inhibited the apoptosis induced by Adpa-Mn (Fig. 5D). At the same time, the Adpa-Mn-induced MDC-labeled particle formation and LC3 conversion were inhibited by NAC (Fig. 5E and F). These results demonstrate that ROS are necessary for the Adpa-Mn-induced apoptotic and autophagic death of HepG2 cells.

### In vivo anticancer activity of Adpa-Mn against a mouse hepatocellular carcinoma xenograft

**Figure 6:**
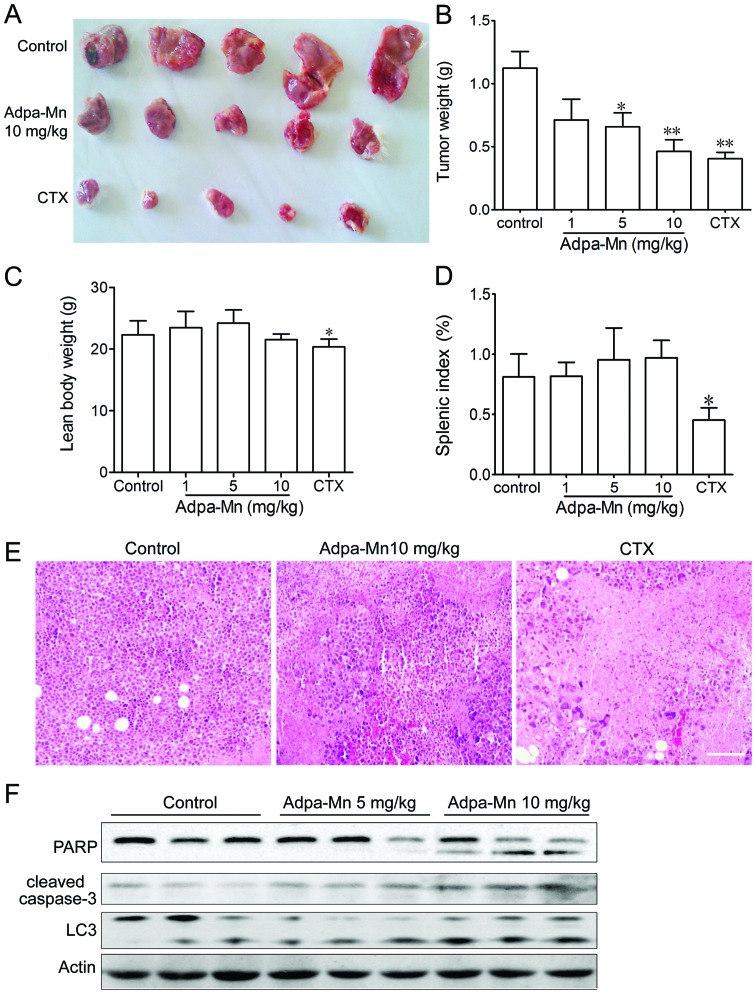
Adpa-Mn inhibits tumor growth *in vivo*. Hepatocellular carcinoma (Hep-A) 1×10^7^ tumor cells (grown in donor mice) were transplanted subcutaneously into the armpits of ICR mice. One day after transplantion, the mice were randomly allocated to either the control or treatment groups, with 10 mice in each group. Drugs were administered intraperitoneally on days 0-9. After the mice were sacrificed, solid tumors were separated. (A and B) Tumors were photographed and weighed. (C and D) Lean body weight and thymus splenic index were calculated. (E) Paraffin-embedded sections of tumor tissues from mice were analyzed by H&E staining. Scale bar, 50 *μ*m. (F) Protein from tumor tissue from mice in each group was extracted and analyzed by western blot analysis. Data represent the means ± SEM. n=10, ^*^p<0.05 and ^**^p<0.01 vs. control.

To examine the antitumor activity of Adpa-Mn *in vivo*, we developed a mouse xenograft model of Hep-A cells in ICR mice. The administration of Adpa-Mn (1, 5, 10 mg/kg, once a day) inhibited tumor growth in the mice in a dose-dependent manner (Fig. 6A and B). The tumor inhibition rate at the dose of 10 mg/kg was 60%, which is comparable to that of 20 mg kg CTX, and H&E staining revealed evident cell death in the tumor tissue (Fig. 6E). Treatment with CTX significantly reduced the body weight and splenic index in the mice, whereas no significant changes were observed in the Adpa-Mn-treated mice (Fig. 6C and D). According to the results *in vitro*, both PARP and caspase-3 activation and LC3 conversion in the tumor tissue from mice were significantly enhanced following treatment with Adpa-Mn, which proved that apoptosis and autophagy were induced (Fig. 6F). These results indicated that Adpa-Mn effectively suppressed tumor growth *in vivo,* while no significant side-effects were observed.

## Disscusion

The success of cisplatin in the treatment of cancer patients suggests that other metal complexes may also be potential drugs in future chemotherapy regimens. In this study, experiments were performed to verify whether the designed manganese compound, Adpa-Mn, can be used as an anticancer lead compound. *In vitro*, Adpa-Mn was demonstrated to be active against various types of tumor cells in a dose- and time-dependent manner. *In vivo*, the growth of mouse hepatocellular carcinoma tumor xeno-grafts was significantly attenuated by Adpa-Mn, which was comparable to the effect of CTX.

The anticancer activity of Mn(II) has, however, been distinctly enhanced by combination with diverse ligands, including chrysin (32,33). For most of these compounds, interactions with DNA involving intercalation or coordinative binding have been demonstrated. However, the knowledge of the precise molecular mechanisms underlying their increased cytotoxic activity against cancer cells remains limited. N-allyl di(picolyl)amine (Adpa) has been shown to be active against the proliferation of cancer cells (34), capable of complexing Cu(II) and inducing cell death by DNA interaction and ROS-mediated autophagy (35,36).

The majority of metal complexes, such as platinum or copper complexes have shown activity for DNA binding and cleavage and the ability to induce cell cycle arrest and apoptosis (36–39). In a previous study of ours, we found that the Adpa-Mn complex exhibited high toxicity against cancer cell lines, but showed weak DNA binding and cleavage activity (34). In agreement with the study that manganese can induce apoptosis in neuronal cells (40), in this study, treatment with Adpa-Mn induced apoptosis, as indicated by nuclei condensation and the appearance of apoptotic bodies, which occurred through the mitochondrial pathway (Fig. 3).

Usually, apoptosis is the major mechanism which destroys cancer cells. A number of chemotherapeutic agents have been designed to kill cancer cells through the induction of apoptosis (41). For example, cisplatin has been reported to induce apoptosis in various types of human tumor cells (42). However, as is known, the decreased effects of anticancer drugs or resistance to apoptosis are becoming a major concern with the long-term use of chemotherapeutic agents. Thus, an alternative form of programmed cell death known as autophagy is becoming increasingly important in cancer therapy (24,43,44). Previously, autophagy was referred to as a physiological process that plays a protective role as cells encounter environmental stresses, such as starvation and pathogen infection (45). It was also classified as type II programmed cell death or autophagic cell death (46). Excessive autophagy can also act as a pro-death mechanism. Rapamycin and its analogs, which induce autophagic cell death by inhibiting mTOR, have been demonstrated to be a potent therapeutic strategy for many tumor types in preclinical clinical studies (47). In this study, we demonstrated that Adpa-Mn induced autophagy, which indeed contributed to its cell death-inducing mechanisms (Fig. 4). The following characteristics of autophagy were observed in the present study: the formation of AVO and the punctate distribution of LC3 and the elevated ratio of LC3-II to LC3-I. Furthermore, we confirmed that the Adpa-Mn-induced cell death was mediated through autophagy: cell death was significantly suppressed by the inhibition of autophagy, by pre-treatment of the cells with various autophagy inhibitors or the transfection of siRNA targeting ATG7 (Fig. 4).

It is well established that mitochondria and ROS play a central role in the process of cell death, including apoptosis and autophagy (48–53). It has been suggested that the mitochondria can regulate the release of proteins inducing apoptosis and autophagy through the excessive generation of ROS and the self-directed induction of mitochondrial permeability transition (MPT), while ROS play several roles in cellular processes, including DNA damage, mitochondrial dysfunction, the activation of signaling pathways and the activation of transcription factors, leading to the upregulation of genes (54). Consistent with these observations, in this study, Adpa-Mn induced mitochondrial dysfunction, including the collpase of mitochondrial membrane potential following the accumulation of ROS. When ROS were scavenged, both the Adpa-Mn-induced autophagy and apoptosis were hampered (Fig. 5) which proved that ROS generation triggered by Adpa-Mn was responsible for the apoptotic and autophagic cell death. However, the association between the apoptosis and autophagy induced by Adpa-Mn warrant further investigations, which may provide some strategies for the regulation of apoptosis/autophagy and drug design.

Taken together, our findings suggest that Adpa-Mn exhibits potent and stable anti-proliferative and cytotoxic activity against diverse tumor types *in vitro,* as well as against tumor xenografts mediated by the ROS-dependent apoptotic and autophagic cell death. Our study thus provides useful insight into the investigation of apoptosis and autophagy in cancer cells and offers a rationale for the development of complexes as effective chemotherapeutic agents against human cancer in clinical settings.

## Figures and Tables

**Table I:** Half maximal inhibitory concentration (IC_50_) of Adpa-Mn in various cancer cell lines.

Cell line	IC_50_ (M)
HeLa	12.3±1.5
HepG2	10.8±0.9
A549	14.2±0.5
MCF-7	6.5±0.7
U251	9.1±0.6
LoVo	10.2±0.4
A875	18.6±0.3
ECA-109	16.2±0.3
