# [Corrigendum] Tumor-targeting novel manganese complex induces ROS-mediated apoptotic and autophagic cancer cell death

**DOI:** 10.3892/ijmm.2026.5968

**Published:** 2026-08-26

**Authors:** Jia Liu, Wenjie Guo, Jing Li, Xiang Li, Ji Geng, Qiuyun Chen, Jing Gao

Int J Mol Med 35: 607-616, 2015; DOI: 10.3892/ijmm.2015.2073

Following the publication of the above article, an interested reader drew to the authors' attention that the COX IV western blot data shown in Fig. 3D on p. 611 were strikingly similar to blots that appeared in another article by the same research group that was published in the journal *Molecular Medicine Reports*. In addition, it was noted that a section of the fluorescence microscopic data shown in Fig. 4A (in the 'Adpa-Mn 20 *µ*M + 3-MA' data panel) bore a strong resemblance to the data shown in Fig. 5A for the 'Adpa-Mn 20 *µ*M' experiment. Thirdly, concerns were raised regarding the tumour images shown in Fig. 6A. Certain of the tumours appeared rather large, although a corresponding scale alongside the tumours was not shown in the figure; in addition, the tumour weights reported in Fig. 6B appeared not to be commensurate with the apparent sizes of the tumours, and Fig. 6B showed quantification of the data for three different Adpa-Mn treatment groups (1 mg/kg, 5 mg/kg and 10 mg/kg), whereas only tumours from the 10 mg/kg group were portrayed in Fig. 6A.

Upon re-examining the raw data belonging to these figures, due to oversights that were made during the final assembly and handling of a large volume of experimental data, the authors realize that some errors were inadvertently made in assembling the data in various of the figures. In Fig. 3, the western blot band for the COX IV loading control was misused, and an incorrect band was included in this figure. In Figs. 4 and 5, certain fluorescence microscopy images were misplaced during the final preparation, leading to the incorrect placement of the data panel in Fig. 4A. Finally, in Fig. 6A, the tumour images for the 1 mg/kg and 5 mg/kg treatment groups had not originally been included, and a scale bar was omitted from Fig. 6A. Concerning the animal experiments, the Editor permitted the authors to repeat the *in vivo* experiments under rigorous conditions, and in the new version of Fig. 6, a complete set of tumour photographs for all groups has been provided, now including a scale bar.

The revised versions of Fig. 3 (showing the correct data for the COX IV western blots in Fig. 3D) and Fig. 4 (showing the correct data for the 'Adpa-Mn 20 *µ*M + 3-MA' panel in Fig. 4A), and the new version of Fig. 6, are shown on the subsequent pages. Note that the errors made during the assembly of these figures did not affect the overall conclusions reported in the paper. All the authors agree with the publication of this corrigendum, and are grateful to the Editor of *International Journal of Molecular Medicine* for allowing them the opportunity to publish this. They also apologize to the readership for any inconvenience caused.

## Figures and Tables

**Figure 3:**
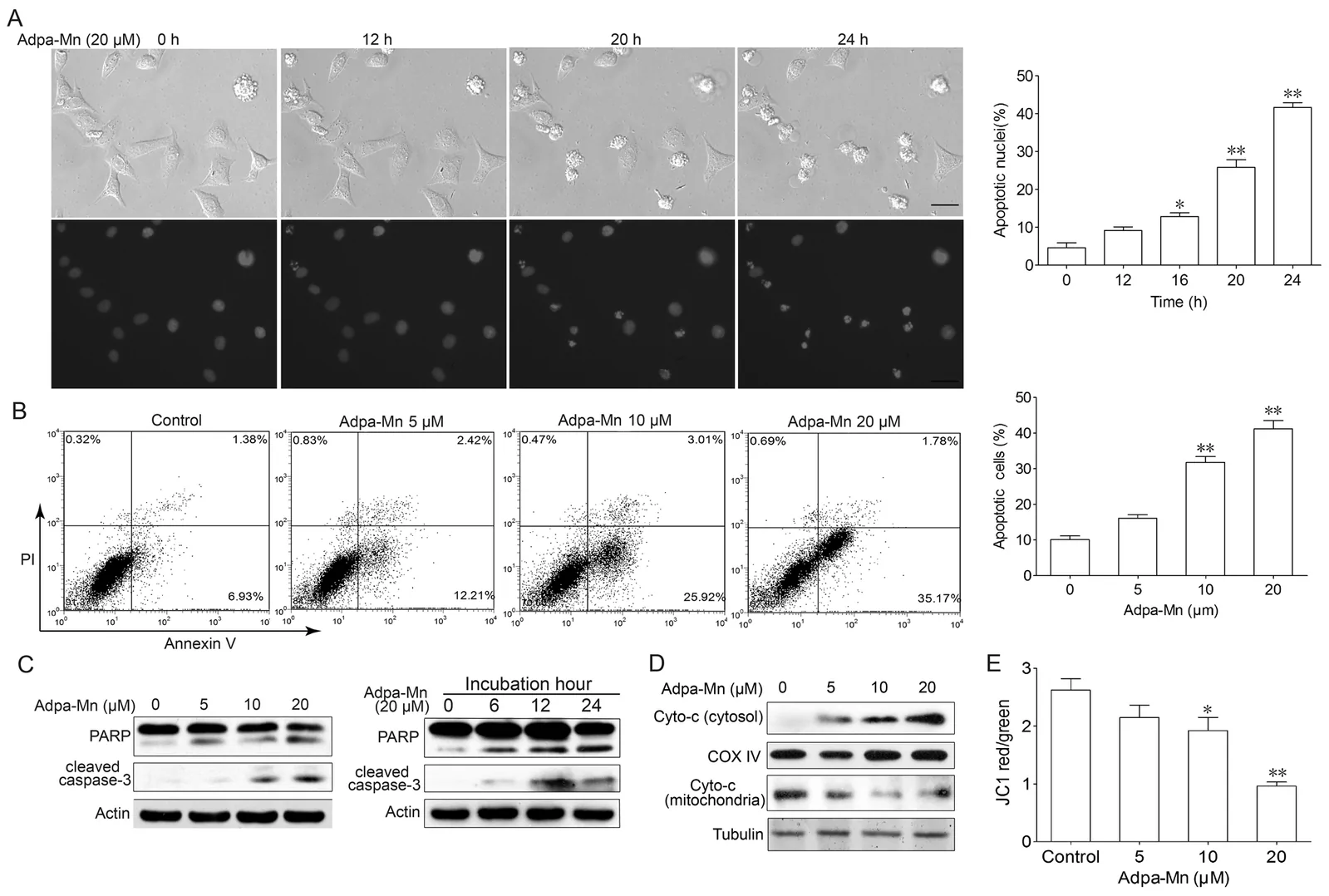
Adpa-Mn induces apoptotic cell death. (A) H2B-GFP-labeled HeLa cells (stable cell line) were treated with Adpa-Man (20 *µ*M) for 24 h, morphology and nuclei were photographed using a Nikon TE2000 microscope with a live cell system. Apoptotic nuclei were counted in 10 fields of vision at each time point and the apoptotic percentage was calculated. Scale bar, 5 *µ*m. (B) HepG2 cells treated with Adpa-Mn were collected and subjected to Annexin V/propidium iodide (PI) staining. (C) PARP and caspase-3 activation were assessed by western blot analysis. (D) The release level of cytochrome c from the mitochondria was examined by western blot analyiss. (E) HepG2 cells treated with Adpa-Mn were collected and subjected to mitochondrial membrane potential analysis. Data represent the means ± SD of 3 different experiments. ^*^p<0.05 and ^**^p<0.01, as compared with the untreated (control) group.

**Figure 4:**
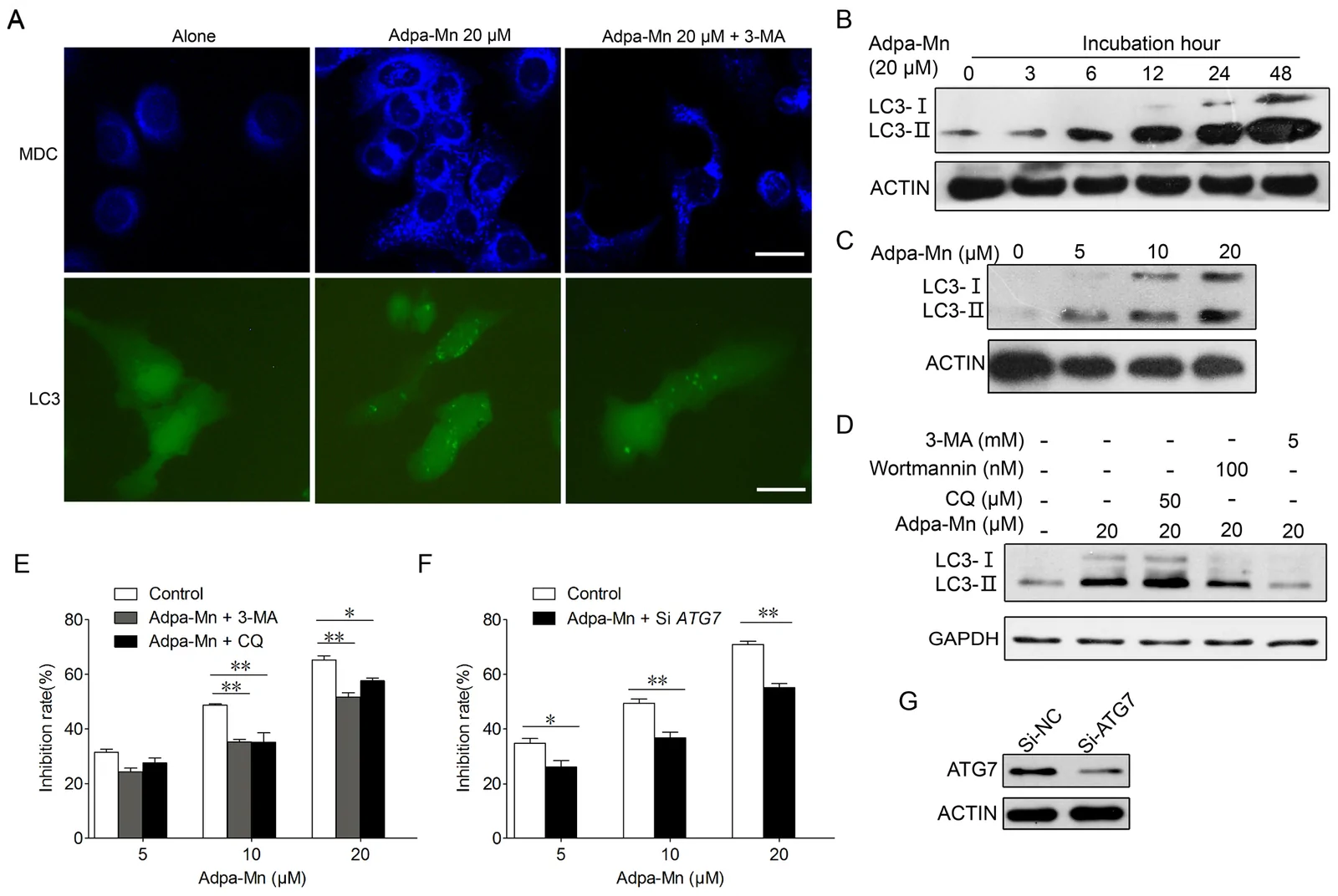
Adpa-Mn induces autophagic cell death. (A) HepG2 cells transfected with GFP-LC3 cDNA were treated with 20 *µ*M Adpa-Mn for 12 h with or without pre-treatment with 5 mM 3-methyladenine (3-MA) for 2 h. The formation of vacuoles containing GFP-LC3 (dots) was examined by fluorescence microscopy. In another set of experiments, HepG2 cells were treated with 20 *µ*M Adpa-Mn for 12 h with or without pre-treatment with 5 mM 3-MA for 2 h and then incubated with 0.05 mM monodansylcadaverine (MDC) for 10 min. The cells were then analyzed by fluorescence microscopy. Scale bar, 5 *µ*m. Protein expression of LC3 in (B-D) was determined by western blot analysis. (B) HepG2 cells were cultured with 20 *µ*M Adpa-Mn for 3, 6, 12, 24 and 48 h. (C) HepG2 cells were cultured with the indicated concentrations of Adpa-Mn for 24 h. (D) HepG2 cells were treated with 20 *µ*M Adpa-Mn for 24 h with or without 3-MA, wartmanin and chloroquine (CQ) pre-treatment for 2 h. (E) HepG2 cells were treated with 20 *µ*M Adpa-Mn for 24 h with or without 3-MA, and CQ pre-treatment for 2 h. MTT assay was used to evaluate the cell death rate. (F) HepG2 cells were transfected with control siRNA or siRNA targeting autophagy-related gene (ATG7). After 48 h, the cells were treated with 0, 5, 10 or 20 *µ*M Adpa-Mn for 24 h, and cell death was measured by MTT assay. (G) The knockdown of ATG7 was confirmed by western blot analysis. Data represent the means ± SEM of 3 different experiments. ^*^p<0.05 and ^**^p<0.01 vs. respective control.

**Figure 6:**
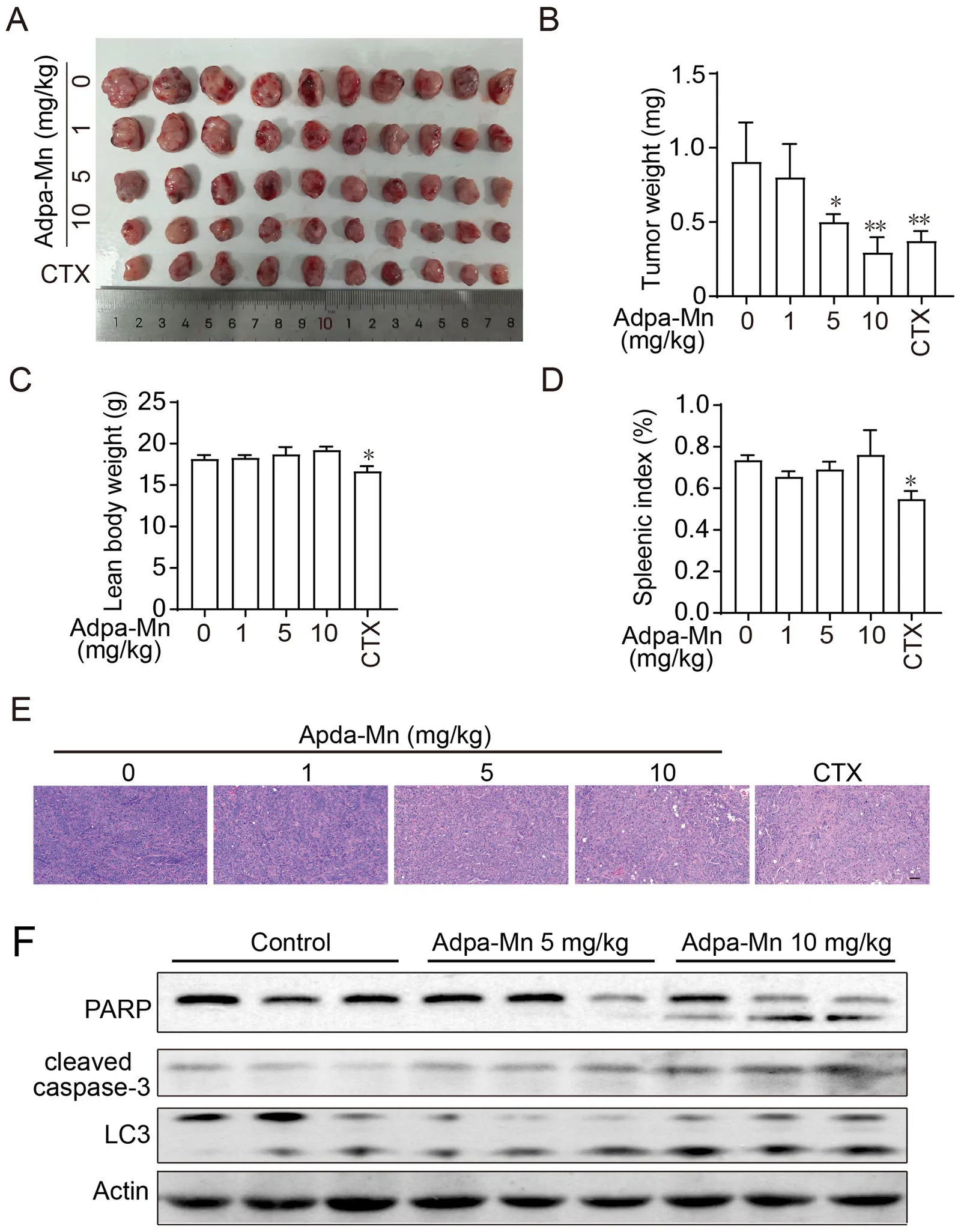
Adpa-Mn inhibits tumor growth *in vivo*. Hepatocellular carcinoma (Hep-A) 1x10^7^ tumor cells (grown in donor mice) were transplanted subcutaneously into the armpits of ICR mice. One day after transplantion, the mice were randomly allocated to either the control or treatment groups, with 10 mice in each group. Drugs were administered intraperitoneally on days 0-9. After the mice were sacrificed, solid tumors were separated. (A and B) Tumors were photographed and weighed. (C and D) Lean body weight and thymus splenic index were calculated. (E) Paraffin-embedded sections of tumor tissues from mice were analyzed by H&E staining. Scale bar, 50 *µ*m. (F) Protein from tumor tissue from mice in each group was extracted and analyzed by western blot analysis. Data represent the means ± SEM. n=10, ^*^p<0.05 and ^**^p<0.01 vs. control.western blot analysis. Scale bar, 5 *µ*m.

